# Molecular Basis of Root Nodule Symbiosis between *Bradyrhizobium* and ‘Crack-Entry’ Legume Groundnut (*Arachis hypogaea* L.)

**DOI:** 10.3390/plants9020276

**Published:** 2020-02-20

**Authors:** Vinay Sharma, Samrat Bhattacharyya, Rakesh Kumar, Ashish Kumar, Fernando Ibañez, Jianping Wang, Baozhu Guo, Hari K. Sudini, Subramaniam Gopalakrishnan, Maitrayee DasGupta, Rajeev K. Varshney, Manish K. Pandey

**Affiliations:** 1 International Crops Research Institute for the Semi-Arid Tropics (ICRISAT), Hyderabad 502324, India; s.vinay@cgiar.org (V.S.); h.sudini@cgiar.org (H.K.S.); s.gopalakrishnan@cgiar.org (S.G.); r.k.varshney@cgiar.org (R.K.V.); 2 Department of Biochemistry, University of Calcutta, Kolkata 700019, Indiamaitrayee_d@hotmail.com (M.D.); 3 Department of Botany, Sister Nibedita Government General Degree College for Girls, Kolkata 700027, India; 4 Department of Life Sciences, Central University of Karnataka, Kadaganchi-585367, India; 5 DBT-National Agri-food Biotechnology Institute (NABI), Punjab 140308, India; 6 Instituto de Investigaciones Agrobiotecnológicas (CONICET-UNRC), Río Cuarto-5800, Córdoba, Argentina; 7 Agronomy Department, University of Florida, Gainesville, FL 103610, USA; wangjp@ufl.edu; 8 Crop Protection and Management Research Unit, United State Department of Agriculture- Agriculture Research Service (USDA-ARS), Tifton, GA 31793, USA; baozhu.guo@usda.gov

**Keywords:** legume, groundnut, peanut, *Arachis hypogaea*, *Rhizobium*, nod factor, crack-entry, root nodule symbiosis, phytohormones

## Abstract

Nitrogen is one of the essential plant nutrients and a major factor limiting crop productivity. To meet the requirements of sustainable agriculture, there is a need to maximize biological nitrogen fixation in different crop species. Legumes are able to establish root nodule symbiosis (RNS) with nitrogen-fixing soil bacteria which are collectively called rhizobia. This mutualistic association is highly specific, and each rhizobia species/strain interacts with only a specific group of legumes, and vice versa. Nodulation involves multiple phases of interactions ranging from initial bacterial attachment and infection establishment to late nodule development, characterized by a complex molecular signalling between plants and rhizobia. Characteristically, legumes like groundnut display a bacterial invasion strategy popularly known as “crack-entry’’ mechanism, which is reported approximately in 25% of all legumes. This article accommodates critical discussions on the bacterial infection mode, dynamics of nodulation, components of symbiotic signalling pathway, and also the effects of abiotic stresses and phytohormone homeostasis related to the root nodule symbiosis of groundnut and *Bradyrhizobium*. These parameters can help to understand how groundnut RNS is programmed to recognize and establish symbiotic relationships with rhizobia, adjusting gene expression in response to various regulations. This review further attempts to emphasize the current understanding of advancements regarding RNS research in the groundnut and speculates on prospective improvement possibilities in addition to ways for expanding it to other crops towards achieving sustainable agriculture and overcoming environmental challenges.

## 1. Introduction

Leguminosae, after Asteraceae and Orchidaceae, the third-largest family of angiosperms and includes many agronomically and economically important crops [1]. The legume family consists of 770 genera and 19,500 species [2]. Papilionoideae, the largest subfamily within the Fabaceae, is divided into distinct phylogenetic clades, namely genistoids, dalbergioids, indigoferoids, milletioids, robinioids, and inverted repeat-lacking clade (IRLC) [3]. Popularly studied model legumes within these clades include *Aeschynomene* and *Arachis* (groundnut) within the dalbergioids, *Glycine* (soybean) and *Phaseolus* (common bean) in milletioids, *Lotus* amongst the robinioids and *Medicago* (alfa-alfa), and *Pisum* (pea) in the IRLC clade.

Legumes are distinct from non-legume species in terms of the nitrogen acquisition by developing root nodules that fix atmospheric nitrogen through symbiotic N_2_-fixing rhizobia. This trait is both ecologically and agriculturally important. Legume rhizobia symbiosis initiated about 58 million years ago [4]. Interestingly, humans would require an extra 288 billion kilograms of fuel to produce the same amount of nitrogen that is fixed by legumes each year through the process of biological nitrogen fixation (BNF) [5]. BNF assimilates atmospheric nitrogen in the form of organic compounds as a sustainable source of nitrogen in an agricultural context. Organically fixed nitrogen can be utilized directly by the plant and, advantageously, it is less susceptible to denitrification, volatilization, and leaching [6,7].

Root-nodule symbiosis (RNS) allows legume plants to house diazotrophic bacteria in an intracellular manner [8]. RNS establishment involves rhizobial invasion through root epidermis, and nodule organogenesis across the root cortical cells. The most common strategy of invasion is through root hair curling and infection thread (a cellulosic tube that allows rhizobial cells to migrate and infect root cells) formation, where the nodule primordia are induced from a distance [9]. Infection Thread (IT) formation is common among temperate legumes such as *Vicia* sp., *Trifolium* sp. and *Pisum* sp. Model legumes *Medicago truncatula* and *Lotus japonicus* also display IT-mediated rhizobial invasion [10,11]. However, there is an alternative mode of rhizobial invasion, crack-entry, where rhizobia enter via an intercellular route at the lateral root base. Approximately 25% of legumes are adapted to ‘crack-entry’, which is a characteristic feature for some subtropical legumes belonging to dalbergoid/genistoid clades such as *Arachis* sp., *Stylosanthes* sp. and *Aeschynomene* sp. [12]. In these crop species, rhizobia directly access the cortical cells to develop nodule primordia, and the infected cells divide repeatedly to form a mature aeschynomenoid nodule in which the core of the infected zone remains separated from uninfected cells [13].

Groundnut, an important crop species belonging to the Fabaceae (Leguminosae) family, subfamily Papilionoideae, is globally one of the major oilseed crops. Importantly, the N_2_ fixation efficiency of groundnut is relatively low as compared to other legume species, thus leaving scope for improvement [14]. Contrary to the model legume plants, the molecular mechanism of root nodule symbiosis is less studied in groundnut. The latest evidences came from recent studies which involved molecular-omics approaches to identify key factors controlling the inception and progress of symbiosis through ‘crack-entry’ in selected legumes including groundnut [15,16]. These studies provided evidence of the interactions of the Nod factor (specific signal molecules secreted by rhizobia) with several signalling and hormonal biosynthesis-related genes during rhizobial infection. However, information is still limited on the ‘crack-entry’ mechanism for legumes belonging to the dalbergioid/genistoid clade, which are basal in their divergence within the Papilionoideae. Therefore, understanding the evolution of nitrogen-fixation by root nodules and the identification of key genes involved in this phenomenon in different legume species is important for sustainable crop production. Within legumes, groundnut can be used as a model crop plant to understand the crack entry mechanism. Further, combining the available knowledge and the molecular explanations from legumes species such as groundnut, *M. truncatula* and *L. japonicus* will help in developing an in-depth understanding of the molecular networks involved in RNS. In this article, we reviewed the advances in research pertaining to symbiotic interaction in a complex ecological system and the progress made in the molecular aspects of RNS, with special reference to groundnut.

## 2. Diversity among Bacterial Strains Associated with Groundnut RNS

Rhizobia are a collection of diazotrophs belonging to the class of α-proteobacteria which includes the genera *Rhizobium*, *Bradyrhizobium*, *Azorhizobium*, *Mesorhizobium*, *Sinorhizobium*, *Methylobacterium*, *Phyllobacterium*, *Ochrobactrum*, *Shinella,* and *Devosia*. Some other nodulating bacteria belonging to the genera *Burkholderia*, *Cupriavidus*, and *Herbaspirillum,* belonging to the class of β-proteobacteria, have been described as well [17]. It is worth mentioning that *Bradyrhizobium* has a basal position amongst all nitrogen-fixing rhizobia, [18] and is reported to be involved in endophytic or symbiotic associations with plant roots [19]. Groundnut forms effective nodules with rhizobia belonging to genus *Bradyrhizobium* [20,21]. A high level of species diversity and fixation efficiency of symbionts was reported from different geographical regions by morpho-physiological and molecular methods [22]. The variability of fixation efficiency is certainly linked to the intrinsic features of each partner and the capacity of the host plants to recognize and retain the compatible partner [23]. This process possibly involves a complex evolutionary phenomenon including the horizontal transfer of symbiotic genes into rhizobia strains and the specific legume genotypes located in various geographic zones [24]. For instance, the 16S/23S analysis of 35 strains from genus *Bradyrhizobium* showed very high similarity (95%–100%) and also confirmed that *Bradyrhizobium* is a major root nodule symbiotic generous for nodule development in groundnut across the world [21,24]. Phylogenetic analysis also revealed the presence of a new subgroup of *Bradyrhizobium,* forming a symbiotic association with groundnut. Recent studies identified novel genospecies of *Bradyrhizobium* involved in nodulation [21,25]. Well-supported groups, obtained based on the intergenic spacer (IGS) sequences, were also retrieved using the *nodC* gene, suggesting *nodC* and IGS regions are generally transmitted vertically. Zazou et al. [26] identified similar infective strains that have been isolated previously from groundnut nodules, but, interestingly, few strains were close to bacteria previously isolated from the roots of purple bush-bean (*Macroptilium atropurpureum*), apple-ring acacia (*Faidherbia albida*), and cowpea (*Vigna unguiculata*). This study also showed that groundnut can be nodulated by *Bradyrhizobium* isolates from soybean [27], or cowpea [28]. However, in Argentina and Morocco, species such as *R. huautlense, R. giardinii*, *R. galegae,* and *R. tropicii*, were found to be associated with nodule formation in groundnut [29,30]; therefore, nodulation in groundnut involves a broad range of *Bradyrhizobium* species.

## 3. Effect of Abiotic Stress on Groundnut Root Nodule Symbiosis

### 3.1. Temperature

Rhizobia symbiosis in legume crops is sensitive to stress, thus affecting the N_2_ fixation and productivity of legume crops [31,32]. Temperature affects the survival of rhizobia and limits RNS. Groundnut typically grows in tropical or semi-tropical areas with soil surface temperatures above 35 °C [33]. The optimal temperature range for growing rhizobia in culture is 28–31 °C and many are unable to grow at 37 °C, including some groundnut symbionts. Heat-shock proteins were produced in *Bradyrhizobium* strain under exposure to 40 °C [34]. In the case of groundnut at 37 °C, it was reported that biomass production of rhizobia (*Bradyrhizobium* sp. ATCC10317, SEMIA 6144 and TAL1371 strains) was slightly reduced, cellular content of low molecular weight oligosaccharides was increased significantly, and neutral glucan synthesis was fully suppressed [35].

Elevated soil temperature affects root growth as well as root hair infection, bacteroid differentiation, and the structure and functioning of the root nodule in *Trifolium subterraneum* [36]. In legumes, optimal temperature varies from 35–40 °C for N_2_ fixation [37]. The influence of root temperature on nodulation and nitrogen fixation by *Bradyrhizobium* sp. forming nodules has been studied in groundnut [34]. The increase in the groundnut root temperature by 37 °C reduced the N_2_ fixation by *Bradyrhizobium* without affecting the nodulation; however, when the root temperature was increased to 40 °C, the nodulation was completely inhibited. This study confirmed that the RNS in groundnut is sensitive to root temperature compared to other legumes because groundnut-infecting Bradyrhizobia species are more sensitive to the increased temperature as compared to the groundnut genotypes.

### 3.2. Osmotic and Saline Stress

Osmotic and saline stress can affect the growth of soil rhizobia by limiting root colonization, infection inhibition, nodule development, and functioning. Rhizobia growing under stress conditions may trigger various changes in biochemical and physiological functioning. In groundnut rhizobia, changes in membrane lipid composition in response to salinity and variation in trehalose content were detected in response to osmotic stress [38,39]. In groundnut, RNS is more sensitive to saline than to osmotic stress [40]. Application of 100 mM NaCl completely inhibited nodule formation on groundnut by *Bradyrhizobium* strains ATCC10317, TAL1000, and SEMIA 6144, and only TAL1371 was capable of inducing nodulating, although the nodule number, size and dry weight were significantly reduced, which altered the N accumulation in shoots. In contrast, groundnut plants subjected to osmotic stress (20 mM PEG6000) showed nodulation patterns similar to control plant [39].

### 3.3. Soil pH

The effect of soil pH on the nodulation process has been extensively studied, due to an increase in the acidic soil area in the world [41]. Angelini et al. [42] demonstrated that the growth of groundnut rhizobia under acidic soil adversely affects their ability to colonize roots and the formation of root nodules. For instance, a reduction in the number of nodules was observed when groundnut genotypes were inoculated with acid-sensitive isolates. The effect was reversed by the addition of 10 mM Ca^2+^, at which point it increased steadily and reached higher values than those obtained at pH 7.0. Interestingly, a decrease in the nodule number was not observed when the groundnut genotype was inoculated with acid-tolerant isolates. These studies suggest that acid tolerant isolates can be used as a source of strains to promote groundnut production in the acidic soil region.

In order to determine their ability to induce nod genes, groundnut root exudates were tested and it was found that all the flavonoids (naringenin, apigenin, chrysin, luteolin, genistein, daidzein) assayed at pH 7 were able to induce the expression of the *nodC* gene, but at different levels. At an acidic pH, very low inductions were observed in acid-sensitive isolates, while acid-tolerant isolates were able to induce the flavonoids in the root efficiently. Further, the exudates released from the groundnut roots grown in an acidic medium can significantly induce *nodC* gene activity [43].

## 4. Processes Involved during Groundnut Root-Nodule Symbiosis

### 4.1. Pre-Infection

Before entry into legume root tissues, the rhizobia colonize near the root surface. Prior to pre-infection signalling, rhizobia start to agglomerate around the root as a microbial biofilm. The bacterial population within the rhizosphere interact mutually through a quorum-sensing system which involves the secretion of homoserine lactone-signalling molecules [44]. Bacterial adhesins and plant cell wall lectins may serve to promote the adhesion of particular classes of bacteria to the root surface [45,46]. The perception of bacterial Nod Factor (NF) typically results in both temporal and spatial distinct morphological, physiological, and molecular responses in roots. This process has been extensively studied in legumes that are infected through root hairs. Rhizobia’s attachment to groundnut roots is dependent on the bacteria growth stage, and the optimal attachment was observed for the bacteria at late log to early stationary phase. Further, Dardanelli et al. [47] reported the involvement of cell surface proteins of *Bradyrhizobium* sp. during RNS. This protein appeared to be a calcium-binding adhesion because cells treated with EDTA were found to be able to bind to adhesin-treated roots, as this protein has similar properties to those reported for rhicadhesin. Most probably, this rhizobia adhesin is involved in the attachment process of rhizobia to the root surface for the establishment of an effective symbiosis during the crack entry of groundnut.

### 4.2. Role of Rhizobial Exopolysaccharides in Root-Nodule Symbiosis

Rhizobial infection for nodule development requires bacterial exopolysaccharides (EPS) in legumes such as *Medicago* sp., *Pisum* sp., *Trifolium* sp., and *Leucaena* sp. [48,49]. Earlier, EPS was considered a critical matrix component of the threads that influence infection-thread penetration. Stacey et al. [50] proposed alternative roles of EPS in the development of determinate (round), or indeterminate (continually elongating), nodules by pointing out the morphological differences in infection threads. Another study by Leigh and Coplin, [51] proposed that EPS act as suppressors of plant defense systems. Interestingly, EPS perception in two nodule ontogenies, determinate and indeterminate, appears to be different. In fact, EPS is essentially required for the establishment of nitrogen-fixation and symbiosis in legumes with an indeterminate type of nodule, whereas plants that develop determinate nodules do not have such a requirement [52,53].

In groundnut, an EPS-impaired mutant was used to understand its role during rhizobia interaction with groundnut roots. The inoculation of groundnut with an EPS-impaired mutant caused the development of fewer nodules, leading to a reduced shoot dry weight and diminished nitrogen content in different organs. Electron-microscopic observation of nodules induced by the EPS-impaired mutant confirmed low nodule occupancy with few bacteroids, and resulted in the formation of empty, non-nitrogen-fixing (Fix^−^) nodules [54]. These results confirmed that EPS mutation affects the molecular process, which controls the establishment of the symbiotic relationship during nodulation. This indicated that rhizobial EPS plays an important role in establishing an effective symbiosis through crack entry in groundnut. Additionally, as infection threads are not formed in groundnut, Morgante et al. [55] proposed that EPS are involved in the evasion of plant defense responses. This was further supported by the fact that, compared to rhizobia, which are enclosed within infection threads, the rhizobia spreading intercellularly are continuously exposed to the defense system of the plant, and thus require the suppression of the plant defense system [51].

### 4.3. Routes of Bacterial Invasion

**Figure 1:**
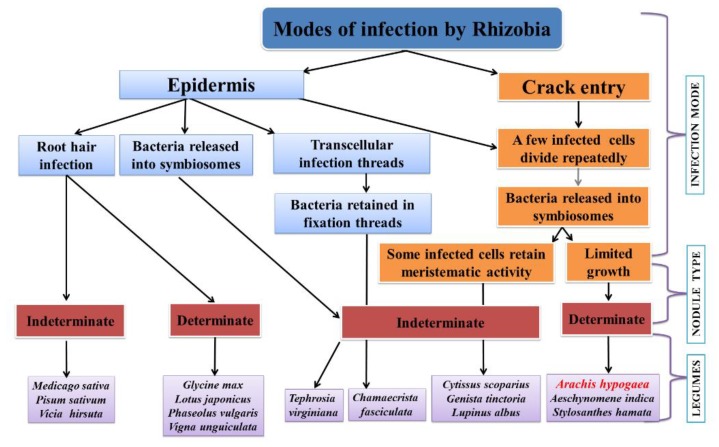
Mode of infection and nodule types among legumes including groundnut (*Arachis hypogaea*).

**Figure 2:**
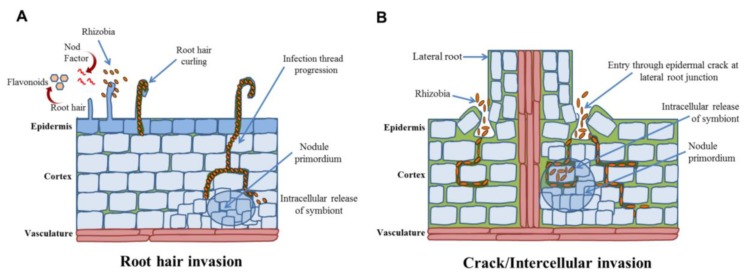
Rhizobial invasion can occur either through root hairs or cracks epidermis. (**A**) Root hair invasion is initiated by rhizobial adhesion to root hairs and root hair deformation. Infection thread initiates from this invasion and allows rhizobial invasion of the cortex. Concomitant with these epidermal responses, cortical cells activate cell division for nodule primordium. (**B**) Crack/intercellular invasion breached the epidermis and rhizobia get direct access to cortical cells.

In legumes, during nodule symbiosis, rhizobial invasion can follow three routes of entry that are host-determined: (1) through root hairs, (2) through wounds, particularly where lateral or adventitious roots occur, and (3) between undamaged epidermal cells. The mode of infection and nodule types among different legumes are shown in Figure 1. Root hair invasion, which led to the formation of an infection thread resulting in the curling of root hair that traps bacteria in the curl, allowing their entry into root hairs through the hydrolysis of cell walls and invagination of the plasma membrane (Figure 2), is the best-studied mechanism involved in root nodulation [56]. Tip growth towards the base of root hairs results in an intracellular infection thread that proceeds through the cortical cells to reach the nodule primordia, where bacteria are released inside cells [9]. Invasion of the cell occurs by accomplishing endocytosis from unwalled infection droplets that originated from the tip of infection threads. Within the host cells, rhizobia are enclosed by a “plant-derived” peri-bacteroid membrane [57,58]. This invasion occurs in members of legume subfamilies Mimosoideae and Papilionoideae, like pea, soybean, vetch, alfalfa, *M. truncatula* and *L. japonicus*.

In the Caesalpinioideae subfamily of legumes, 23% of the members are known to nodulate [59] and most of them belong to the tribe Caesalpiniae, but, exceptionally, the genus *Chamaecrista* belonging to the *Cassiae* tribe also forms nodules, which suggests that nodulation might have independently evolved in this genus [60]. Interestingly, caesalpinioid legumes possess a prototypic attribute, which shows infection progression delimited within conspicuous intercellular “fixation threads”. Moreover, the symbiotic rhizobia remain confined within these infection thread resembling structures and fix nitrogen, but never release inside plant cells attaining the typical bacteroid form, as observed in other legumes [61]. In *Mimosa scabrella,* rhizobia penetrate directly between undamaged epidermal cells by the disruption of the middle lamella of radical cell walls and invade host cells through IT-like structures [62]. Another invasion mode that occurs via natural wounds caused by splitting of epidermis and the emergence of young lateral or adventitious roots is known as crack entry. This invasion occurs in a few sub-tropical legumes like *Arachis* sp., *Neptunia* sp., *Sesbania* sp., *Aeschynomene* sp., and *Stylosanthes* sp. (Table 1). In S. *rostrata* and *Neptunia* sp., invasion leads to the formation of intercellular infection pockets [63,64], which give rise to the formation of intracellular infection threads. However, in A. *hypogaea*, *Stylosanthes* sp., and *Aeschynomene* sp., the structures similar to infection threads have never been observed [65]. In addition, a different mechanism of invasion was described for the symbiosis between *Chamaecytisus proliferus* (tagasaste) and *Bradyrhizobium* sp. The entry of *Bradyrhizobium* to the roots involves the formation of infection threads that later abort and, instead, rhizobia use the crack entry mode to infect the host and for nodule formation. No successful infection threads have been observed at any stage of host infection, and the later rhizobia penetration in the periphery of the nodule primordia occurs intercellularly [66].

In groundnut, the rhizobial invasion mechanism differs from other legumes. Bradyrhizobia penetration of root and cortex, as well as spreading within the nodules occurs, without infection threads formation and involves intercellular invasion/ crack-entry [4]. Peculiarly, tufts of thick-walled multicellular rosette hairs or axillary hairs can be seen in the lateral root junctions in groundnut, having some unassigned function [75]. Surprisingly, these axillary hairs also show curling upon infection with cognate rhizobia in the case of *Arachis* and *Stylosanthes*. Although *Bradyrhizobium* cells were seen to be entrapped within typical “shepherd’s crooks” structures on the apex of some axillary hairs, this never led to infection thread progression like the root hairs of other legumes [65] (Figure 2). Instead, Bradyrhizobia enters the root through the middle lamella between two adjacent axillary hair cells, a place where the cell wall seems to be loosely constructed. After penetration, rhizobial dissemination occurs intercellularly by separating cortical cells at the middle lamella. Some of the axillary root hairs are associated with large basal cells, which are the first to become infected by bradyrhizobia. These large invaded basal cells divide repeatedly to form a determinate nodule where the core infected zone is not intermixed with uninfected cells [67].

### 4.4. Structural Features of Nodule

**Figure 3:**
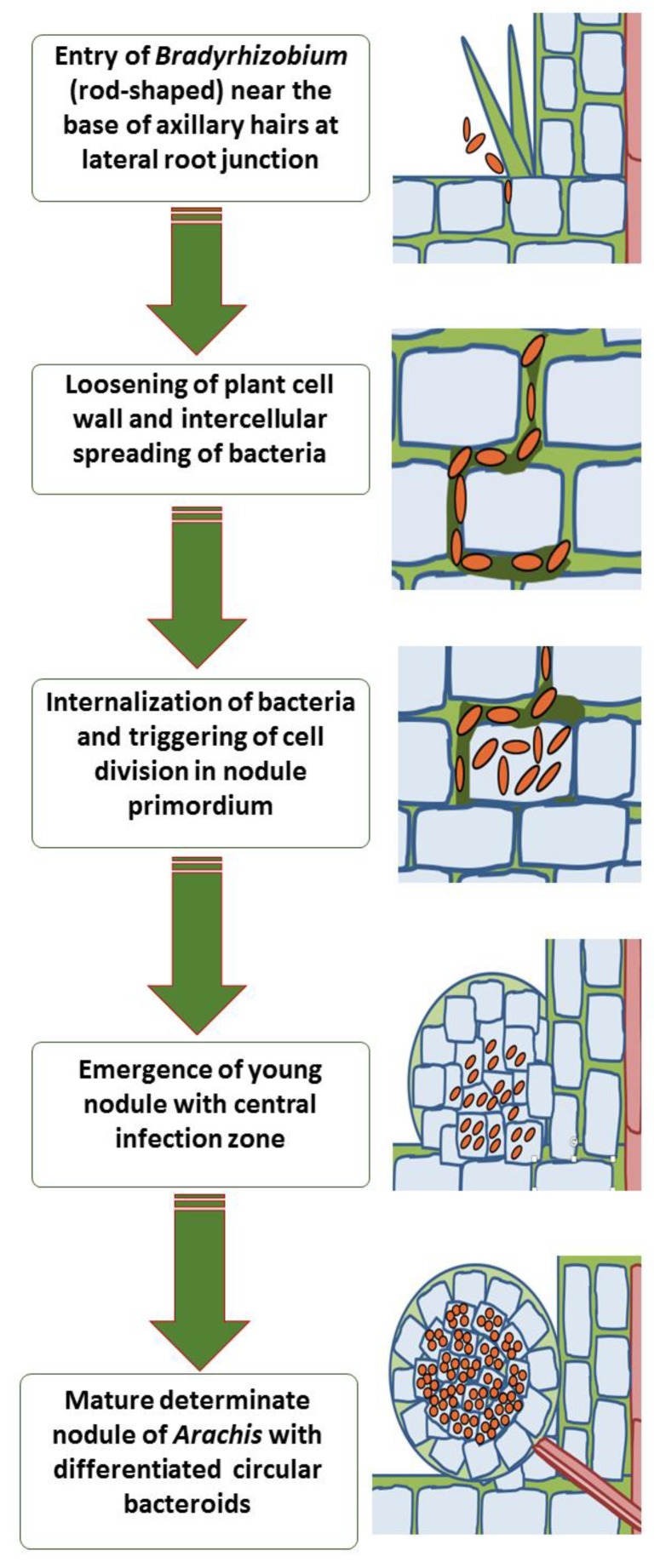
Successive stages of nodule development in groundnut and “crack-entry” invasion of *Bradyrhizobium*.

The successful accomplishment of nodule organogenesis after bacterial internalization leads to an active nodule. Groundnut nodules can be morpho-anatomically assigned under aeschynomenoid-type, which develop at the lateral root emergence axils and are determinate in nature, are oblate-spheroid in shape with 1–5 mm diameter, and are devoid of interspersed uninfected cells in the central infection zone [76]. After invaded cortical cells stop dividing, the previously rod-shaped Bradyrhizobial cells differentiate into swelled-up, spherical bacteroids that become solitarily encapsulated into peribacterial membrane sacs. Nodules show bacteroids tightly packed in the cortical cell cytoplasm, most of which show a central vacuole and peripherally delimited nucleus. The bacteroids are enclosed singly in peri-bacteroidal membrane sacs [77]. Furthermore, ultra-structurally, four types of inclusion bodies are exclusively present in the groundnut nodule: microbodies, oleosomes, electron-dense bodies, and proteinaceous inclusions. Oleosomes (lipid accumulations) and microbodies are found outlining the peribacterial membrane and dense bodies are situated at the peribacteroid space, adjacent to bacteroids [78] (Figure 3).

## 5. Molecular Basis of Root Nodule Symbiosis Initiation in Groundnut

Root nodule symbiosis (RNS) essentially involves a complex molecular dialogue between the plant and symbiotic microbe, ultimately leading to successful symbiosis [79]. After the establishment of symbiotically functional nodules, differentiated bacteria (bacteroids) convert molecular dinitrogen into ammonium ions [80]. As the first signal of this molecular communication, leguminous roots start to release flavonoids that accumulate in the rhizosphere. These compounds activate the bacterial transcriptional regulator protein *NodD*, which, in turn, induces the transcription of other nodulation genes (*nod*, *nol*, and *noe* genes), whose products are involved in the synthesis and secretion of main rhizobial nodulation signals called Nod factors (NF) or lipo-chitooligosaccharides (LCOs) [81].

With some exceptions in temperate legumes like *Aeschynomene* sp., *Sesbania* sp., and *Arachis* sp., very few studies have addressed the molecular aspects of crack entry/intercellular invasion. Groundnut was predominantly reported to be nodulated with *nodABC*-bearing *Bradyrhizobium* strains, but was also found to be nodulated by *nodABC* lacking *Bradyrhizobium* strain Btai1 [82], which suggests that NFs might not be indispensable in the RNS of groundnut [83,84]. Therefore, groundnut may harbor two different modes of RNS: (i) NF-dependent and (ii) NF-independent. In the NF-dependent mode of RNS, Nod factor molecules are required to trigger the cellular divisions for nodule primordium development [83]. Groundnut root junctional cracks are intercellularly infected by rhizobia, and typical aeschynomenoid nodules are formed by repeated divisions of an intracellularly infected cell. Considering these facts, two alternative models for NF perception and infection have been previously proposed. According to the first model, NFs are required for the intracellular infection of the basal cortical cell that forms the nodule primordium. Alternatively, the second model proposes that rhizobia can accommodate intracellularly without NFs, but to reinitiate the meristematic activity of the infected cell, NFs play a significant role. However, the exact degree of colonization of the Bradyrhizobial *nodC* mutant (whether it has an inter- or intra-cellular mode) is still not clearly understood [83], and neither of the two possibilities can be firmly ascertained.

## 6. Components of Symbiotic Signalling Pathway in Groundnut 

### 6.1. AhNFR1 and AhNFR5

The molecular perception of NFs induces symbiotic signalling that allows the establishment of nitrogen-fixing root nodule symbiosis. In the model legume *Lotus japonicus*, NFs are perceived by two LysM domain-containing Receptor-Like Kinases *LjNFR1* and *LjNFR5*, which form a heteromeric complex [85]. Against a groundnut background, Ibanez et al. [86] reported a *LjNFR1* as an orthologue of *AhNFR1* and a *LjNFR5* as an orthologue of *AhNFP* with phylogenetic confirmation. EST contigs, which showed significant similarities to *LjNFR1*, contained extracellular LysM domains in the predicted protein sequences, having characteristic CXC motifs in the interspacer domains situated between LysM1-LysM2 and LysM2-LysM3. Also, PCR-based strategies retrieved amplicon with a high identity with *LjNFR5 like RLKs*, which was found to encode the extracellular region of *AhNFP.* A three-dimensional homology model of the LysM2 domain NFP was also represented, which showed the conserved proximity of glutamine and isoleucine residues assigned to NF binding. However, transcriptional analysis of post infection root samples showed no significant induction of *AhNFP* [86]. Previous reports showed the failure of *Bradyrhizobium* SEMIA *nodC* mutants in nodulation, suggesting the NF dependence of groundnut [83]. Interestingly, Noisangiam et al. [82] mentioned NF-independent nodulation by *Bradyrhizobium* BTAi1, indicating NF dependence as well as independence in groundnut. To conclude, studies thus far have ascertained the presence of NFR in groundnut, but the NF dependence of groundnut nodulation seems to be partner-specific for different strains of *Bradyrhizobium*. 

### 6.2. AhSYMRK

Symbiotic Receptor Kinase (SYMRK) is an indispensable component of a common symbiotic pathway that amplifies the NF-perceived signal [87]. In groundnut, *AhSYMRK* is conserved, structurally composed of an N-terminal signal peptide, followed by a NORK sequence-like domain or Malectin-like domain, three LRRs (leucine-rich repeats), a transmembrane domain and the cytoplasmic kinase domain followed by a C-terminal tail. *AhSYMRK* has been reported to be a dual-specific serine threonine tyrosine (S/T/Y) receptor kinase, which auto-phosphorylates on its “gate-keeper” (Y670) residue [88]. In the biological context, *AhSYMRK* was capable of cross-species complementation in *M. truncatula* TR25 (SYMRK null background) and rescued nodulation, showing complete intracellular accommodation of *Sinorhizobium*. RNA interference (RNAi) of SYMRK was also found to downregulate symbiotic genes (unpublished). Interestingly, the autoactivated kinase domain (KD) of *AhSYMRK* was found to excessively activate nodule organogenesis in the absence of rhizobial stimulus when overexpressed in TR25. Regardless, *AhSYMRK* KD overexpression could not auto-nodulate groundnut, because prior bacterial invasion was presumably needed for nodulation [89]. Intriguingly, substituting gatekeeper Tyr with residues like Phe or Ala significantly affects the restoration of proper symbiotic features in TR25 where rhizobial invasion could not surpass the epidermal–cortical barrier resulting in halted infection patches at the nodule apex. These findings strongly indicated the necessity of an optimally phosphorylated state of SYMRK for aiding rhizobial infection progress and ensuring intracellular invasion in the nodule cortex [90]. Contextually, how such epidermal–cortical regulations can be relevant in groundnut has yet to be understood.

### 6.3. AhCCaMK

Ca^2+^/calmodulin-dependent protein kinase (*LjCCaMK*/*MtDMI3*) is indispensable for the calcium-spiking signal response of the common symbiosis pathway (SYM pathway), which leads to rhizobial invasion through the epidermis towards the cortex and triggers nodule organogenesis [10,91,92]. *AhCCaMK* was found to have a conserved sequence, showing a catalytic kinase domain having subdomains I to XI of Ser/Thr kinases, a junctional domain, and a CaM-binding domain, finally followed by a visinin-like domain with three calcium characteristic binding motifs. *AhCCaMK* could successfully complement null TRV25 *M. truncatula* plants (Unpublished). Notable changes observed in *AhCCaMK* RNAi plants were: (a) a considerable delay in nodulation and a drastic reduction in nodule count was seen in RNAi roots compared to control; (b) the aberrant distribution of *Bradyrhizobium* with scattered pockets containing fewer or no symbiosomes, and an abundance of bi-nucleated cells were noted. Further, a scanning electron microscopy (SEM) study revealed pleomorphic, geometrically irregular bacteria in the symbiosomes; (c) the acetylene reduction assay confirmed reduced nitrogenase activity in the RNAi nodules in comparison with control transformed nodules [93]. All of these observations indicate the active role of *AhCCaMK* in nodule organogenesis and endosymbiont dissemination and proper symbiosome formation during nodule development. 

### 6.4. AhCYCLOPS

CYCLOPS is a transcription factor working in the SYM pathway, which is phosphorylated by CCaMK [94]. Activated CYCLOPS binds to the CYCLOPS-responsive cis-elements placed in the promoter region of NIN and ERN1 for their transcriptional activation [95,96], or signal transduces to other transcription factors like NSP1, NSP2 and RAM1 [97]. Structurally, CYCLOPS contains a variable N-terminal regulatory domain, followed by an activation domain (AD) and two nuclear localization sequences (NLS1 and NLS2) ending with a C-terminal conserved coiled-coil region in the DNA binding domain [95]. Das et al. [98] characterized *AhCYCLOPS*, unraveling different facets of its role in nodulation. Under the expression of a native promoter, *AhCYCLOPS* was fully capable of cross-species complementation in *M. truncatula ipd3-1* and *ipd3-2* background. The silencing of *AhCYCLOPS* through RNAi in groundnut resulted in the delayed inception of nodulation compared to control plants. However, the infection zones of these RNAi nodules were uniformly infected, but the Bradyrhizobia were rod shaped (undifferentiated) without functional spherical bacteroids. Furthermore, the silencing of the *AhCYCLOPS* gene significantly affected the expression of several other important symbiotic gene homologues, such as *AhCCamK*, *AhHK1*, *AhNIN*, and *AhENOD40* in roots. The expression of *AhCCaMK* was unaltered but *AhHK1* expression showed an almost 1.5-fold increase, but a drastic decrease was noted, in the transcription level of *AhENOD40*, while *AhNIN* showed a moderate decrease. Conclusively, the attenuation of *AhCYCLOPS* affected the differentiation of rhizobia, probably due to down-regulation of symbiotic signalling components. 

### 6.5. AhHK1

Cytokinin is sensed by the cyclase/histidine kinase-associated sensing extracellular (CHASE) domain-containing receptors like cytokinin response1 (*MtCRE1*) and *Lotus* histidine kinase1 (*LHK1*) [99,100]. Cytokinin-triggered phosphorelay through these histidine kinases (HK) is essential for nodule organogenesis [101,102]. In groundnut, the *histidine-kinase1* (*AhHK1*) gene was predicted to contain the extracellular cytokinin-sensing N-terminal CHASE domain flanked by two transmembrane domains, a cytosolic kinase domain followed by a C-terminal receiver domain. The RNAi of *AhHK1* was shown to down-regulate type-A response regulators like *AhRR5* and *AhRR3,* along with symbiotic genes like *AhNIN* and *AhENOD40*. There was also a significant reduction in nodulation in *AhHK1-RNAi* roots and the developed nodules were inactive. Moreover, the infected nodules showed a high mitotic index and the presence of rod-shaped, undifferentiated rhizobia within spherical symbiosomes. Notably, elevated expression of the meristem maintenance factor *Wuschel-Related Homeobox 5* (*WOX5*) was found, which could be correlated to the undifferentiated condition of *AhHK1-RNAi* nodules. Overall, all these findings indicated that *AhHK1*-orchestrated cytokinin signalling is required for both nodule inception and their progression from proliferating to a differentiated cell fate during development [103]. 

Functional genomics has provided new insights into a wide number of candidate genes and transcription factors involved in root nodule symbiosis in legumes [15,16] (Table 2). The functional characterization of these genes will add new layers to the existing understanding of RNS.

## 7. Signalling and Phytohormone Pathways Involved during Nodulation in Groundnut

Several reports of transcriptomic studies show the biosynthesis of hormones and the genes behind its activation or degradation being differentially expressed upon NF treatments. We summarized the effects of phytohormones on nodulation in Table 3.

### 7.1. Auxin

Several findings link Nod Factor (NF) signalling to auxin transport, which is inhibited by flavonoids. NF application or *S. meliloti* infection inhibits auxin transport from shoot to root at 24 h, as well as regulating the expression of some *MtPIN* auxin efflux transporter genes in an *MtCRE1*-dependent manner [102,104]. Moreover, *MtCRE1*-dependent pathways also control the accumulation of flavonoids in *M. truncatula* roots upon infection, and flavonoid application can rescue the *Mtcre1* nodulation phenotype. These data suggest that NF-induced cytokinin signalling triggers flavonoid induction and the subsequent inhibition of polar auxin transport. The resulting accumulation of auxin initiates cortical cell division and nodule organogenesis.

Available evidences suggest that NFs regulate the expression of auxin signalling genes. NF application induces transcription factors encoding genes *MtARF16a* and *MtPLETHORA3* in root hairs related to auxin, while several *ARF* genes are downregulated in NF-treated root hairs after 24 h [105,111]. However, these genes were found to be induced by auxin in roots of *M. truncatula* [112]. It indicates that the perception of NF could lead to the accumulation of auxin which, in turn, activates some specific auxin signalling genes which are reported to control cell divisions or IT formation in legumes [105]. No information is available thus far in groundnut to provide clarity as to the role of auxin transport in groundnut nitrogen fixation.

### 7.2. Cytokinin

Various studies documented the crucial role of cytokinins (CKs) as key regulators of nodule organogenesis [99,100] and IT formation [111,113]. A comparative analysis between groundnut and the model legumes highlighted the predominance of CKs and ethylene signalling pathways during nodule formation. It was found that the two-component CK receptor *Histidine kinase 1* (*HK1)* plays a central role in nodule organogenesis in both groundnut and other model legumes [99,101,102,103]. During nodule development, the *AhHK1*, *LjHK1* (*LHK1*), and *MtCRE1* have a similar pattern of expression, but *AhHK1,* a downstream effector, showed an altered pattern of expression during groundnut symbiosis. Expressions of CK-responsive TFs such as *type-B RR* like *MtRR1, MtNSP1,* and *MtbHLH476* have a distinct expression pattern in groundnut in comparison to model legumes. The distinct role of CK signalling during groundnut nodulation is in accordance with the previous report, where the silencing of *AhHK1* resulted in delayed nodulation associated with nodule differentiation [103].

Rhizobia–legume interaction activates the common symbiosis pathway (SYM-pathway) that recruits CK signalling for the induction of nodule primordia in the cortex [114]. CK-signalling causes local auxin accumulation at the site of incipient nodule primordia by modulating the expression of auxin transporters [102,104]. These phytohormonal signals and the SYM-pathway together reprogram the cortical cells and regulate their division, ultimately building a nodule primordium for the endocytic accommodation of the symbionts [115,116]. Loss-of-function mutations of *LHK1* (lhk1-1 allele, formerly known as hit1)/*MtCRE1* (cre1-1/2) lead to a strong reduction in nodulation [101,102], suggesting that CK-dependent phosphorelay through these receptor histidine kinases is essential for nodule organogenesis. Further, gain-of-function mutation of *LHK1* (*snf2*) is sufficient to induce nodule primordia in the absence of rhizobia, demonstrating that CK signalling is sufficient to initiate this developmental process [100]. A recent study unraveled the role of CK signalling in groundnut [103]. This study provided insight on the silencing (RNAi) of putative CK receptor *Histidine Kinase1*(*AhHK1*) resulting in a decrease in nodule number, indicating that CK signalling, mediated through this receptor, is important for the inception of nodule primordium.

CK’s role in nodule differentiation was first reported by Plet et al. [102], but whether this involves factors like *retinoblastoma-related protein* (*RBR*), which restrains proliferation to promote the differentiation of cells [117], and the Wuschel-related homeobox5 (*WOX5*) meristem maintenance factor [118], which are functionally conserved in root nodule meristems, is a subject for future investigation [103]. Further, some evidence suggests that NFs induce CK production, which initially regulates nodule organogenesis, and then rapidly activates negative feedback on NF signalling and infection processes.

### 7.3. Gibberellins

NFs can rapidly induce the biosynthesis of bioactive gibberellin (GA) in root hairs, which consequently triggers a negative feedback leading to both downregulation of NF signalling and the activation of GA catabolic enzymes [106,112,119]. Increasing evidence also shows that bioactive GAs negatively regulate nodulation in both determinate and indeterminate nodules [106,119]. Thus, NF-induced GAs could help fine-tune NF signalling and rhizobium infection during symbiosis.

### 7.4. Ethylene

The ethylene response factor (ERFs) plays a crucial role by regulating cell division and differentiation during nodule development [107,120]. Earlier genetic and physiological studies highlighted the key regulatory role of ethylene during early symbiotic processes [108,121,122,123]. Groundnut transcriptomics highlighted the upregulation of several *AP2-domain* and symbiotic orthologues of *ERF1* during nodulation [15,16]. In *L. japonicus, LjERF1* is a positive regulator of nodulation and downregulates the expression of defense gene *LjPR-10* during symbiosis [107]. Intriguingly, the high expression of *ERF1* in groundnut is associated with the high expression of *PR-1s,* indicating that the ethylene signalling network is differently recruited during symbiosis. The expression of the master regulator of ethylene signalling, *EIN2,* was significantly high in groundnut, and the expression of a negative regulator of nodulation, *EFD,* was distinctly different from model legumes. Vernie et al. [120] observed that the differential role of ethylene signalling during crack-entry nodulation was because an EFD (for the ethylene response factor required for nodule differentiation) appears to be involved in an ethylene-independent feedback inhibition and regulates the expression of cytokinin response during nodulation.

### 7.5. Abscisic Acid

Abscisic acid (ABA) is a meythyl–pentadienoic compound involved in seed maturation, dormancy, and drought stress response pathways [124]. ABA inhibits lateral root development in an auxin-independent manner and governs root architecture, taking cues from nutritional signals [125]. Interestingly, non-legumes like *Arabidopsis* respond to ABA by decreasing lateral root density (LRD) and legumes respond to ABA by elevating LRD. Furthermore, increasing LRD by ABA was found to be associated with the formation of root nodules in the non-legume actinorhizal plant *Casuarina glauca*. Hence, this suggests that increased lateral root formation in ABA response is pivotal in the case of nodule formation [109]. Studies in *M. truncatula* have indicated that ABA can influence both nodule formation and bacterial infection. ABA negatively regulates early gene expressions of *RIP1* and *ENOD11*, possibly through the regulation of the Nod factor signalling pathway. It was also shown that ABA drastically suppresses the cytokinin-orchestrated induction of *ENOD40* and *NIN* in *sta-1*, a mutant sensitive to ABA [110]. Thus, examination of the role of ABA in nodulation is important to enhance the groundnut nitrogen use efficiency in order to reduce the yield gap. 

## 8. Utilization of Existing Knowledge to Enhance N_2_ Use Efficiency by Groundnut and Cereals

### 8.1. Application of Transgenic Approaches and Genome Editing

**Figure 4:**
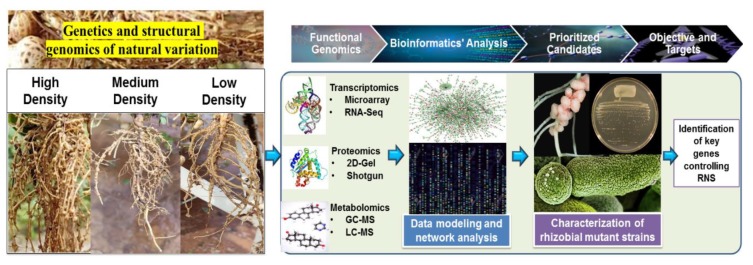
**Deployment of molecular-omics approaches will help in the characterization of important genes in RNS.** First, the investigated natural nodule variation in groundnut is shown, then the functional genomics technologies, and the integrative data analysis used to prioritize candidate genes. Finally, genes important for various stages of symbiosis are identified and validated.

Identifying suitable combinations of candidate genes from the symbiotic pathway is a continual process and relevant efforts are in progress to enhance nitrogen fixation through a transgenic approach [126]. With the advancements in transcriptomics and proteomics, data modelling and targeted selection, the search for candidate symbiotic genes has been efficiently bypassed. Domain swapping and chimeric approaches have also been made in the case of symbiotic pathway components like SYMRK [127]. With the advent of newer technologies like genome editing, manipulating genome structures has become easier. Genome editing tools like CRISPR/Cas9 were employed to knockout *leghemoglobin* (*Lbs*) genes in *L. japonicus*, and uncovered their synergistic roles in symbiotic nitrogen fixation [128]. This study concluded that *Lbs* acts additively in nodules and the lack of *Lbs* results in early nodule senescence. It also provided insight into the reprogramming of the gene expression network in the absence of *Lbs*, probably as a result of an uncontrolled free O_2_ concentration [128]. Various genomics approaches and biotechnology efforts in this direction will help us to learn more about the molecular basis of nodulation at the genomic/proteomic/metabolomic level, which would effectively translate into major gains in research and contribute to more sustainable agriculture (Figure 4).

### 8.2. Context of Advanced Bioinformatics in Groundnut Research

Bioinformatics plays an inevitable role in current plant science because the amount of biological data grows exponentially, leading to a parallel growth in the necessity for data management tools and methods for visualization, integration, modelling, and prediction [129]. In this context, groundnut has a fairly large and complicated allotetraploid genome (2n = 4x = 40, 2800 Mb), which imposes a great challenge for its whole-genome sequencing attempts [130]. Alongside the advancement of next-generation sequencing technologies such as 454 pyro-sequencing and Illumina sequencing, the transcriptomic data of groundnut has been rapidly gathered in genome databases. To exemplify, PeanutDB is one such public genomic database that integrates groundnut transcriptome data, including 32,619 contigs each accompanying EC, KO (KEGG Orthology), and GO (Gene Ontology) functional annotations and tabulated SSRs, SNPs and other genetic polymorphisms [131]. Another widely used database is PeanutBase, affiliated with the Legume Federation, serving as an international community resource for peanut crop improvement. Some features of this database include (i) inclusive data collection templates for genetic maps, markers, QTLs, traits, and publication data, which also encourages researchers to contribute individually generated datasets; (ii) PeanutBase has used the MAKER-P [132] annotation pipeline, in which polypeptide sequences from the annotations of soybean, common bean, and *Medicago* genomes were used, in addition to available expressed sequence tags (EST) data from the wild *Arachis* diploids and cultivated tetraploid [133]. Analyses gained from all of these bioinformatics databases can positively lead to the methodical genetic engineering of groundnut and holistic crop improvement. 

### 8.3. Strategies to Extend Nitrogen Fixation to Non-Legumes

Understanding the strategies used by different legume species to discriminate between and select the best microbial partners in their natural habitat is important for the improvement in nitrogen fixation efficiency in legumes. An ambitious goal is to transfer the symbiotic nitrogen fixation machinery to economically relevant non-legumes such as cereal crops [134,135]. As cereals also contain the common symbiotic pathway (CSP) genes working towards arbuscular mycorrhizal (AM) colonization, the engineering of oxygen-limited nodule-like structures in cereal roots can also be attempted. To modulate symbiotic activity in cereal crops, nodule-independent endophytic association with nitrogen-fixing microorganisms through crack entry or epidermal invasion is also a prospective area of research. Studies have shown the effectiveness of novel metabolites in the selection of beneficial microbial populations in the rhizosphere [136], and plants can be genetically modified to exert specialized metabolites that would selectively enhance the competitiveness of engineered nitrogen-fixing super-microbes in the rhizosphere. 

## 9. Current Menace of Inorganic Fertilizers—Key Challenges

Synthetic fertilizers are expensive and cause environmental pollution, which ultimately gives rise to problem such as eutrophication due to nitrate accumulation in fresh-water bodies. Alarmingly, visionless farming practices are widespread in Southern Asia. Volatilizations of inorganic fertilizers have led to the emission of tremendous amounts of ammonia, which would disturb human health in the long run. Anthropogenic activities have destabilized the nitrogen cycle and also contributed to approximately two thirds of the annual flux in reactive nitrogen species (Nr) into the atmosphere [137]. Ammonia, despite having a short lifetime and limited emission height, in the transport of deposited NH_4+_ from one ecosystem to another, could lead to widespread ecosystem pollution [138]. Statistical studies revealed that 11 countries from South Asia have cumulatively applied ~65% of the global total inorganic manures during 2000–2014. China and India together have contributed ~77% of total inorganic nitrogen application and ~75% of total NH_3_ emission among these countries within the mentioned time-span [139]. Therefore, symbiotic bacterial nitrogen fixation is the best alternative way to boost nitrogen availability and utilization; it is one of the central goals of crop improvement research. 

## 10. Enhancement of Biological Nitrogen Content in Soil by Intercropping with Groundnut

Intercropping is a traditionally advantageous agricultural practice that ensures efficient usage of growth resource nutrients and water in the soil [140]. The combinatorial intercropping of shade-tolerant, non-climbing legumes such as groundnut, soybean, and cowpeas, with cereal crops like maize, sorghum, and millet, and fiber crops like cotton, greatly enhances overall yield compared to singular farming [141,142]. Moreover, legume intercropping with grains can help in reducing weed competition, minimizes soil erosion, and fortifies the organic nitrogen pool. Therefore, such organic practices can reduce the use of synthetic N-fertilizer and herbicides [143]. A lot of reports have exhibited an increment in gross yield after groundnut cultivation. Legumes like groundnut reportedly provide an equivalent of 60 kg N_2_/hectare to succeeding non-legume crops. Relevant to this, wheat cultivation followed by groundnut caused a higher grain yield than wheat cultivation followed by pearl millet. Furthermore, groundnut intercropping with sorghum minimized the inorganic nitrogen fertilizer requirement by 30-84 kg N/hectare [144]. Chu et al. [145] reported that rice/groundnut intercropping increased rice grain yield in low nitrogen soil, by the virtue of the improved organic nitrogen supplement provided by the preceding groundnut batch. Therefore, crop rotation with groundnut can lead to an increased supply of biologically fixed nitrogen, which can, in turn, minimize environmental pollution caused by inorganic fertilizers.

## 11. Harnessing the Genetic Variation through Conventional and Molecular Breeding for Improving Nitrogen Fixation

Selection and breeding have strengthened the concept of N_2_ fixation for decades [146,147]. Nevertheless, the information on genetic variation for nodulation and N_2_ fixation is meager or largely absent [148,149]. In recent years, the collection of germplasm resources, innovation of breeding theory and technology, and marker-assisted breeding have played an important role in accelerating breeding. Strengthening the research and utilization of germplasm resources in a systematic way, with desirable characteristics, will help to identify useful traits for N_2_ fixation selection programs. In addition, due to the availability of tetraploid reference genomes for cultivated groundnut [150,151,152] and high-density genotyping assays [153], these can further accelerate genomics studies, leading to the discovery of associated genes and markers for deployment in breeding, to improve the N_2_ fixation ability of popular varieties. In order to harness the modern breeding technologies, the diverse set of germplasm lines need to be properly phenotyped for nodulation and its component traits under multiple environments and soil textures. It is also equally important to establish a relationship between nodulation efficiency and yield in addition to the estimation of residual N_2_ availability for the next cropping season. Therefore, it is important to understand all the above-mentioned factors related to N_2_ fixation in groundnut in order to exploit the available genetic variability for developing improved varieties with a high N_2_ fixation ability as well as a cost-saving possibility.

## 12. Summary

Groundnut is an important source of food, feed, and edible oil. Its ability to enter into a symbiotic relationship with nitrogen-fixing rhizobia has a strong impact on the environment and agriculture. Improving the nodulation and nitrogen fixation processes are seen as pivotal steps towards enhancing agricultural sustainability, nutrient recycling, soil biodiversity, ecosystem services, and even food security. Therefore, it is important to gain an in-depth understanding of the symbiotic pathways of groundnut, whose mode of bacterial colonization is similar to endophytic colonization, among the non-legume crops, so the transfer of nitrogen fixation machinery can take place with minimal tweaks. With the advancement of technologies, insights into the functional studies of genes from different legumes have been significantly increased, which will help to elucidate the mechanism of nodulation, and also provide candidate genes which can be used for re-decorating genomes of non-legumes to introduce nodulation and N_2_ fixation in them, to enhance productivity and food security.

## Figures and Tables

**Table 1:** List of legumes exclusively showing ‘crack-entry’ mode of infection, with their systematic affiliations, the names of rhizobial partners and the specific features of nodules (legume phylogenetic clades are mentioned within brackets; MCC-Mimoseae–Cassieae–Caesalpinieae).

‘Crack-entry’ Legumes	Systematic Position within Leguminosae (Fabaceae)	Symbiotic Partner Rhizobia	Distinctive Features of Nodule	Reference
*Arachis hypogaea*	Sub family: Papilionoideae Tribe: *Dalbergieae* (Dalbergioid)	*Bradyrhizobium* sp.	Aeschynomenoid type, oblate-spheroid, root junctions with axillary hairs	[67]
*Sesbania rostrata*	Sub family: Papilionoideae Tribe: *Sesbanieae* (Robinioid)	*Azorhizobium caulinodans*	Aeschynomenoid affinities, spherical, ‘open basket’ nodule meristem.	[68]
*Stylosanthes guianensis*	Sub family: Papilionoideae Tribe: *Dalbergieae* (Dalbergioid)	*Bradyrhizobium stylosanthis* *Rhizobium* sp. Strains: CIAT1460, CB2126, CB1650.	Aeschynomenoid type, oblate, root junctions with axillary hairs.	[69,70]
*Aeschynomene* *afraspera*	Sub family: Papilionoideae Tribe: *Dalbergieae* (Dalbergioid)	*Bradyrhizobium* sp.	Aeschynomenoid type, spheroid, root and stem nodules.	[12]
*Neptunia natans*	Sub family: Mimosoideae Tribe: *Mimoseae* (Mimosoid, MCC)	*Devosia riboflavina*	Mimosoid, unbranched- elongated, aquatic infection environment.	[71]
*Mimosa pudica*	Sub family: Mimosoideae Tribe: *Mimoseae* (Mimosoid, MCC)	*Burkholderia phymatum, Cupravidus taiwanensis*	Mimosoid, unbranched- elongated, broad symbiont range.	[72]
*Chamaecrista fasciculata*	Sub family: Caesalpinioideae Tribe: *Cassieae* (MCC)	*Burkholderia tuberum,* *Rhizobium tropici* *Bradyrhizobium frederickii*	Caesalpiniod type, rigid, hemispherical with fixation threads.	[73,74]

**Table 2:** Summary of the transcriptomics-based identification of candidate genes and transcription factors involved in root nodule symbiosis in legumes.

RNS Progress	Key Genes /TFs	Functional Description
Bacterial Recognition	*NFR1/LYK3*	Initiate host response
	*LYR3*	Recognition of symbiotic signals
	*EPR3*	Role in Recognition of Nod factor and host-symbiont compatibility
Early signalling and SYM pathway	*CASTOR*	Encode putative ion channel protein
	*CNGC*	Plays a role in symbiotic calcium oscillations in SYM pathway
	*CYCLOPS*	DNA-binding transcriptional activator induces nodule development
Early Transcription factor	*NIN, NSP2, and ERF1*	Role in transcriptional reprogramming for initiation of root nodule symbiosis
	*ERN1*	Controls rhizobial infection
Infection	*ARPC1*	Encode heptameric ARP2/3 nucleator—essential for the intracellular accommodation of rhizobial bacteria
	*CERBERUS*	Role in infection thread formation, growth, and differentiation of nodules
Cell division	*HK1/CRE1*	Role in nodule organogenesis
	*bHLH476*	Plays a role in cytokinin pathway which positively regulates symbiotic nodulation
Nodule regulation	*EIN2*	Plays a key role in plant–microbe interaction
	*AP2*	Transcriptional regulator of symbiotic nodule development
	*Cytokinin oxidase/dehydrogenase 6*	Maintaining cytokinin homeostasis during root and nodule development
	*CLE13*	Role in systematic autoregulation of nodulation (AON) pathway that negatively regulates nodule number
	*F-box/kelch-repeat protein*	Long-distance regulation of legume–rhizobium symbiosis
	*Protein kinase superfamily protein*	Role in infection and nodule development
	*RWP-RK family protein*	Key regulators of nitrogen responses and of gametophyte development

**Table 3:** Summary of phytohormones and their regulatory effects on nodulation.

Plant Hormones	Overall Influence on Nodulation	Mechanism of Regulation	Reference
Cytokinin	Positive	● Ligand for HK1, essential for cortical signalling ● Delimits polar auxin transport during growth of nodule primordia	[101]
Auxin	Positive	● Required in nodule meristem for cortical division ● Regulated by NF signalling	[104,105]
Gibberellin	Negative	● Nod factor triggered negative feedback maintenance	[106]
Ethylene	Negative / Positive	● Downregulates defense response gene *Lj* Pr-10 ● Downregulates early genes of symbiotic pathway	[107,108]
Abscisic acid	Negative / Positive	● Increases lateral root density (LDR) in legumes ● Negatively regulates ENOD 11 and RIP1	[109,110]
